# The effect size of rs521851 in the intron of MAGI2/S-SCAM on HADS-D scores correlates with EAT-26 scores for eating disorders risk

**DOI:** 10.3389/fpsyt.2024.1416009

**Published:** 2024-12-05

**Authors:** Daria Pinakhina, Evgeny Kasyanov, Grigory Rukavishnikov, Andrey K. Larin, Vladimir A. Veselovsky, Alexander Rakitko, Nikholay Neznanov, Alexander Kibitov, Galina Mazo, Mykyta Artomov

**Affiliations:** ^1^ V.M. Bekhterev National Medical Research Center for Psychiatry and Neurology, Saint-Petersburg, Russia; ^2^ Lopukhin Federal Research and Clinical Center of Physical-Chemical Medicine, Moscow, Russia; ^3^ Genotek Ltd., Moscow, Russia; ^4^ Pavlov First St. Petersburg State Medical University, St. Petersburg, Russia; ^5^ Institute for Genomic Medicine, Nationwide Children’s Hospital, Columbus, OH, United States; ^6^ Department of Pediatrics, The Ohio State University College of Medicine, Columbus, OH, United States

**Keywords:** GWAS, MAGI2, depression, HADS-D, EAT 26

## Abstract

An association between the *MAGI2* (*S-SCAM*) intron variant rs521851 and depression symptoms, as measured by the depression subscale of the Hospital Anxiety and Depression Scale (HADS-D), has been recently reported. The role of *MAGI2* in depression has been linked to disruptions in the gut–brain axis. In this study, we investigated the association between rs521851 and HADS-D scores in an independent cohort of 380 individuals, consisting of 238 patients with an ICD-10 diagnosis of depression and 142 healthy controls. The original association was replicated in the patient cohort but not in the control group. Further analysis revealed that the effect size of rs521851 on HADS-D scores was moderated by Eating Attitudes Test 26 (EAT-26) scores. In participants with an EAT-26 score of ≥20, the effect size of rs521851 on HADS-D was more than 20 times greater compared to those with an EAT-26 score of <20. These findings successfully replicate the original association signal for *MAGI2* and HADS-D, and highlight the role of *MAGI2* in gut–brain interactions.

## Introduction

Major depressive disorder (MDD) has been associated with an elevated incidence of cardiovascular disease (1, 2), diabetes (3), autoimmune (4), and gastrointestinal conditions (5). These findings underscore the significance of recognizing the interconnection between the genetic basis of MDD and somatic diseases. One of the genes hypothesized to mediate the link between gastrointestinal conditions and MDD is *MAGI2*, also known as *S-SCAM* (synaptic scaffolding molecule) (6).

*MAGI2* is one of the membrane-associated guanylate kinase proteins, which play a crucial role in spatial organization of presynaptic and postsynaptic compartments essential for neuronal communication (7). The gene is also involved in tight junction assembly supporting intestinal barrier function (8, 9). This dual functional profile is reflected in its association patterns involving both psychiatric and gastrointestinal conditions.

*MAGI2* associations were found in MDD, response to antidepressants, and hippocampal atrophy—a frequent neuroimaging feature in MDD (10–13). The initial suggestive association between the variants in *MAGI2* and MDD was observed by Coleman et al., 2020 (14) when investigating a cohort of trauma-unexposed individuals from the UK Biobank, with the signal peaking at rs535355 (p = 7 × 10^−7^). Further exploration of the role of *MAGI2 (S-SCAM)* in MDD revealed a genome-wide significant signal for an association with the Hospital Anxiety and Depression Scale depression subscale [HADS-D (15)] score at rs521851 in the same locus in an independent cohort (6). However, the rs521851 association was not replicated in other large-scale GWAS .

Multiple lines of evidence have connected the HADS-D-based depression to inflammatory bowel disease (IBD) (16) and irritable bowel syndrome (IBS). Polygenic risk scores for IBD have been significantly associated with depression identified by HADS-D, and genes associated with HADS-D scores were found to overlap significantly with the differentially expressed genes in the sigmoid colon mucus of IBS-C patients (6, 17). The microbiota–gut–brain axis has been established as a key regulator in IBS (18). Recent studies also emphasize the importance of the gut–brain axis in the pathogenesis of IBD (19). Taken together, these findings suggest a link between HADS-D-based depression and gut–brain axis dysregulation.

One of the factors, which can undermine the replication of GWAS findings across cohorts, is the heterogeneity of the studied conditions (20, 21). Investigating the relationship between the strength of GWAS associations and other clinical characteristics could, therefore, not only provide insights into the pathogenic mechanisms of the studied variants but also improve the robustness of GWAS findings (22). Motivated by this, we aimed to examine the strength of the association between rs521851 and HADS-D in an independent heterogeneous cohort and its relationship with other clinical features. We also utilized the IEU OpenGWAS (23, 24), a catalog of previously reported GWAS results, to further clarify the functional characteristics of rs521851 based on its association profile. The study cohort included four sub-cohorts enrolled between 2019 and 2022 comprising 238 patients with ICD-10-diagnosed depression and 142 individuals without psychiatric disorders. We successfully replicated the association of the MAGI2 variant rs521851 with HADS-D among the patients. Notably, the association between rs521851 and HADS-D scores was particularly strong among patients at high risk for eating disorders. A significant moderating effect of eating disorder risk, measured by the Eating Attitude Test 26 (EAT-26) (25) score, on the relationship between rs521851 and HADS-D scores was observed across the entire cohort. These results align with the previous findings on the role of *MAGI2* in the gut–brain axis disturbances associated with depression.

## Methods

The study was approved by the Independent Ethics Committee at the V.M. Bekhterev National Medical Research Center for Psychiatry and Neurology in 2019 (protocol #7 from 22 June 2018).

### Cohort collection and phenotyping

This pilot cross-sectional, multicenter study was performed under the supervision of the Russian National Consortium for Psychiatric Genetics (RNСPG, http://rncpg.org). Recruitment of subjects for the study was performed in several centers in the Russian Federation (**Supplementary Data Sheet 1**, Research Centers).

Participants were recruited between 2019 and 2022 at the study centers encompassing patients from both outpatient and inpatient services. The entire cohort, totaling 380 participants, included 4 sub-cohorts based on the interview year, comprising 79, 121, 153, and 27 participants for 2019, 2020, 2021, and 2022 respectively (**Supplementary Figure 1A**).The data analysis was conducted in 2023.

### Diagnostic procedures

At the time of enrollment, all study participants underwent a structured clinical interview based on ICD-10 criteria to confirm clinical diagnoses and identify comorbid psychiatric disorders. The control group consisted of individuals without any psychiatric conditions, as determined through diagnostic interviews. The study included patients aged 18 years or older who met the diagnostic criteria for major depressive disorder. Exclusion criteria are detailed in the **Supplementary Data Sheet 1**, Exclusion Criteria. Participants were assessed using a variety of psychometric tools, including the HADS, EAT-26, Columbia—Suicide Severity Rating Scale (C-SSRS) (26), Hypomania Symptom Checklist (HCL-32) (27), and Temperament and Character Inventory (TCI) (28). Additionally, anthropometric and sociodemographic data, along with the age of disease onset, were recorded (**Supplementary Data Sheet 1**, Psychometric Research Tools; **Supplementary Table 1**).

### Genetic data generation

Genotyping was performed in two centers: Lopukhin Federal Research and Clinical Center of Physical–Chemical Medicine and Genotek Ltd. laboratory. In the first center, DNA was extracted using the MagMAX™ Microbiome Ultra Nucleic Acid Isolation Kit and KingFisher™ Purification System (Thermo Fisher Scientific, USA) according to the manufacturer’s protocol. The DNA was subsequently quantified on Qubit 4 fluorometer by Quant-iT dsDNA BR Assay Kit (Thermo Fisher Scientific, USA). In the second center, DNA extraction was performed with QIAamp DNA Mini Kit (Qiagen).

Genotyping of samples was performed using the Illumina Infinium Global Screening Array-24 v3.0 beadchips on the iScan, Illumina in both centers. Alternative allele frequencies did not differ significantly between the centers for all variants (**Supplementary Figure 2**).

Samples with a call rate less than 95% were removed. Variants with a call rate less than 95%, minor allele frequency <0.01, and showing Hardy–Weinberg equilibrium test p < 1 × 10^−6^ were also excluded. Additional genotypes were imputed using the 1000 genomes phase 3 reference panel using Beagle v5.4. The variants with dosage R-squared DR2 < 0.7 were eliminated. rs521851 passed the variant filtration procedure. The final cohort for analyses included 380 samples with high-quality genotypes for rs521851 and HADS-D phenotyping performed. rs521851 alternative allele frequency was similar in the groups of samples that have been genotyped in both centers (0.91 and 0.92).

### Replication analysis

The study cohort was assembled over an extended period. Therefore, to study the heterogeneity in HADS-D scores and HADS-D depression status (defined as HADS-D score ≥ 11) within the cohort, we divided it into several sub-cohorts, based on the interview year. Further, we evaluated the variability in HADS-D scores and depression status through linear and logistic regressions, respectively. The pairwise differences in HADS-D scores between sub-cohorts were examined using the Mann–Whitney test, and the overall differences among all sub-cohorts were evaluated using the Kruskall–Wallis test.

The association between rs521851- and HADS-based depression status was examined in each sub-cohort using Fisher’s exact test. A meta-analysis across all four sub-cohorts (spanning the years 2019–2022) was conducted and in the sub-cohorts of 2020–2022 (the subset of the sub-cohorts without significant heterogeneity). The association was assessed using the Mantel–Haenszel test. For healthy participants, constituting 37% of the cohort, the association was tested without distribution into sub-cohorts by interview year due to the lack of observations (specifically, the absence of subjects with one alternative allele and HADS-D ≥ 11), utilizing Fisher’s test.

In each sub-cohort, a linear regression was conducted to examine the association between rs521851 and HADS-D score. Meta-analyses for the association were carried out across all sub-cohorts spanning the years 2019–2022 and specifically for the sub-cohorts of 2020–2022 employing the fixed-effects model. A singular test using linear regression was executed for the entire patient sub-cohort.

All tests for the associations were performed for a one-tailed hypothesis based on the directionality of the effect for the originally described association (beta = −0.54 for the G allele).

### Analysis of heterogeneity sources in patient sub-cohorts and their moderating effects on the association between rs521851 and HADS-D scores

We conducted a comparison between the sub-cohort of patients enrolled in 2019 and the remaining patients examining all phenotypes reported in **Supplementary Table 1** using the Mann–Whitney test. Following Bonferroni correction, seven phenotypes showed significant differences between the patient groups (**Supplementary Table 1**). Notably, the EAT-26 subscales “dieting” and “bulimia” scores exhibited significant correlations with the EAT-26 total score, and the C-SSRS lifetime suicidal ideation score significantly correlated with the C-SSRS total lifetime risk score. For further analysis, the total scores of the corresponding questionnaires were utilized for these features.

The remaining four features underwent testing for moderation effects on the rs521851 and HADS-D scores in the entire cohort using linear regression. Given the significant moderation relationship of rs521851 on EAT total scores in their effect on HADS-D, we investigated whether EAT-26 scores could account for the observed heterogeneity in the meta-analysis of patient sub-cohorts. This was done through meta-regression analysis incorporating mean EAT-26 scores per sub-cohort.

In further exploring the impact of alterations in eating behaviors measured by EAT-26 on the association between rs521851 and HADS-D, we assessed the Pearson correlation between the effect size (beta) of the association and the threshold EAT-26 score. The findings indicated an association between the total EAT-26 score threshold and the absolute effect size of the association. Subsequently, we examined the rs521851–HADS-D score association specifically in participants with eating disorder risk, as measured by EAT-26 (EAT-26 ≥ 20), utilizing linear regression.

### Examination of rs521851 associations in IEU GWAS catalog

We searched IEU OpenGWAS catalog (24) for associations between rs521851 and phenotypes containing the substring “depress” with the same direction of effect (using a one-tailed hypothesis). Then, we performed a search for the associations with the variant without taking effect directionality into account (with threshold p < 0.05).

### Statistical analysis tools

The analysis scripts were written in python and R programming languages, the package statsmodels v0.13.0 (29) was used to perform regression analyses, the R package stats v4.2.1 (30) was used to perform Fisher and Cochran–Mantel–Haenszel tests, metafor v4.2.0 (31) package was used for the meta-analyses (including meta-regression), scipy v1.4.1 (32) package was used for correlational analysis, and ieugwasr v0.1.5 (24) package was used to search for associations in the IEU GWAS. The packages ggplot2 v3.4.2 (33), seaborn v0.11.2 (34), matplotlib v3.5.2 (35), statannotations v0.4.3 (36), and supervenn (37) were used for data visualization. Pandas v1.0.3 (38), numpy v1.21.0 (39), tidyverse v2.0.0 (40), and dplyr v1.1.2 (41) were used for data manipulation. Hail v0.2 (42) was used for genotyping data processing. Ref (43). was used to process the Illumina microarray data. Beagle v5.4 (44) was used for genotype imputation.

## Results

The study cohort comprised 380 individuals, including 238 patients with depression diagnosed with ICD-10 criteria and 142 healthy individuals without any mental disorders, enrolled between 2019 and 2022 (mean age 31 years, 68% of females). DNA was extracted from all subjects, and microarray genotyping was performed.

**Figure 1:**
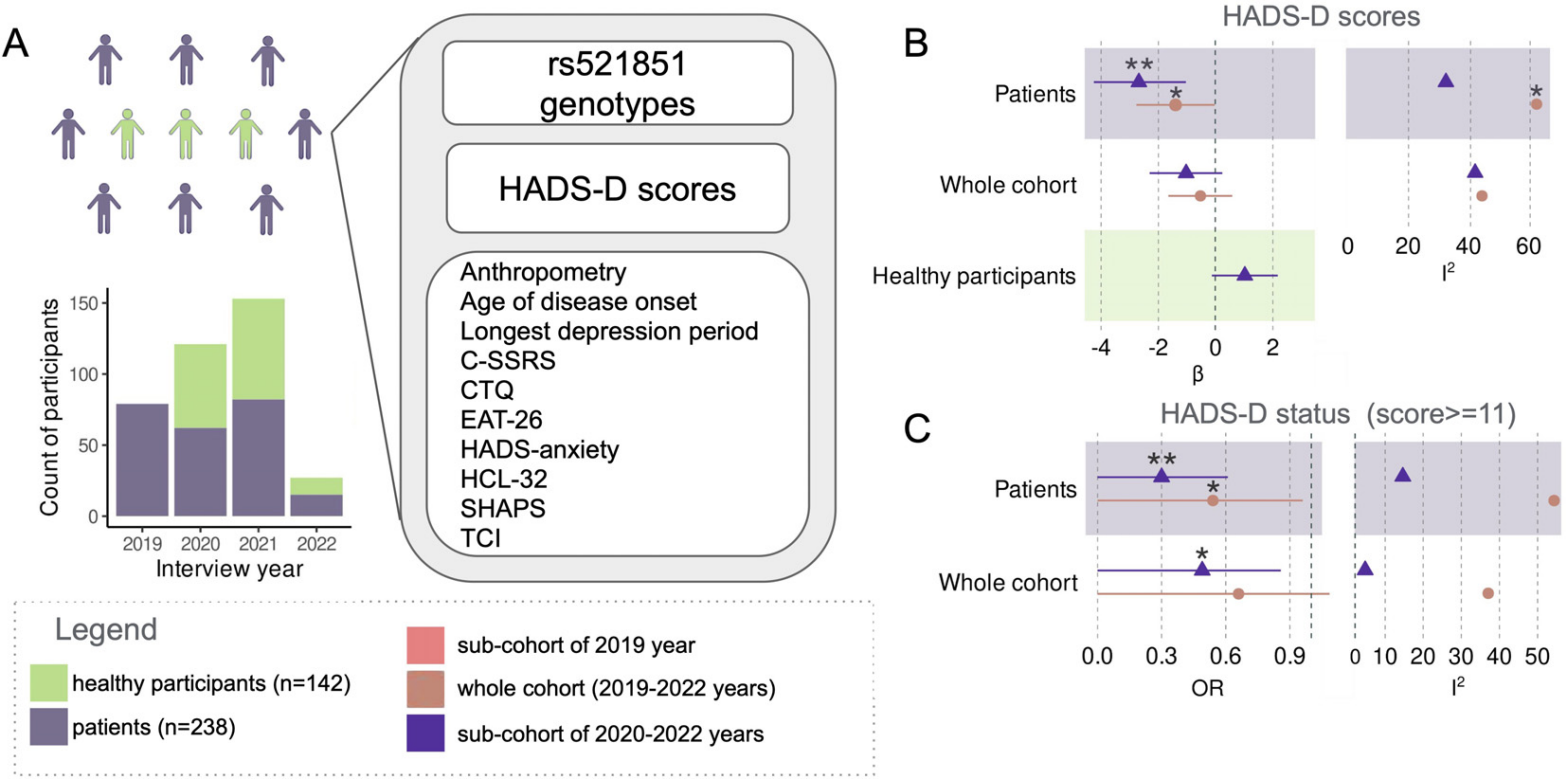
Cohort composition and study design scheme **(A)**. Meta-analysis of the effect of rs521851 variant on HADS-D scores: effect sizes (β) and heterogeneity (I2) **(B)**. Meta-analysis of the effect of rs521851 variant on HADS-D status: effect sizes (β) and heterogeneity **(C)**. *p<0.05, **p<0.01.

Given the prolonged collection period of the cohort, our initial focus was on assessing the homogeneity of the dataset. We investigated the association of HADS-D scores and HADS-D depression status [HADS-D ≥ 11 (45)] with the enrollment year across the entire cohort. Notably, the 2019 sub-cohort, comprising solely patients with ICD-10 depression, exhibited significantly higher HADS-D scores compared to the sub-cohorts of other years (p = 1.3 × 10^−8^; Kruskal-Wallis; **Figure 1A**, **Supplementary Figures 1A, B**).

Subsequently, our objective was to replicate the association between rs521851 and HADS-D scores. However, the meta-analysis for the entire cohort, including both patients with diagnosed depression and healthy controls, did not yield significant results. Upon the exclusion of the 2019 sub-cohort, the variant demonstrated an association with HADS-D status (p = 0.026—Mantel–Haenszel, one sided, OR = 0.49). Importantly, the I^2^ statistic that measures data heterogeneity decreased from 37.29% to 4.64% (**Figures 1B, C**; **Supplementary Table 2**).

The variant exhibited associations with both HADS-D depression status (p = 0.048; OR = 0.54; Mantel–Haenszel, one sided) and HADS-D scores (p = 0.045; beta = −1.40; one-sided t-test, fixed-effects model) in the meta-analyses of depression patient sub-cohorts. The inclusion of the 2019 patients led to an increase in heterogeneity in the meta-analysis of the association with HADS-D score to a significant level (p = 0.048; Q test) and also elevated it in the analysis for HADS-D status (p = 0.087; Q test). The associations were more robust in the meta-analyses of the three remaining patient sub-cohorts after excluding the 2019 data (p = 3.8 × 10^−3^; beta = −2.68 one-sided t-test, fixed effects model—for HADS-D scores; p = 5 × 10^−3^; OR = 0.30 Mantel–Haenszel, one sided—for HADS-D status. **Figures 1B, C**; **Supplementary Table 2**). Notably, no genetic associations were observed among the healthy participants.

**Figure 2:**
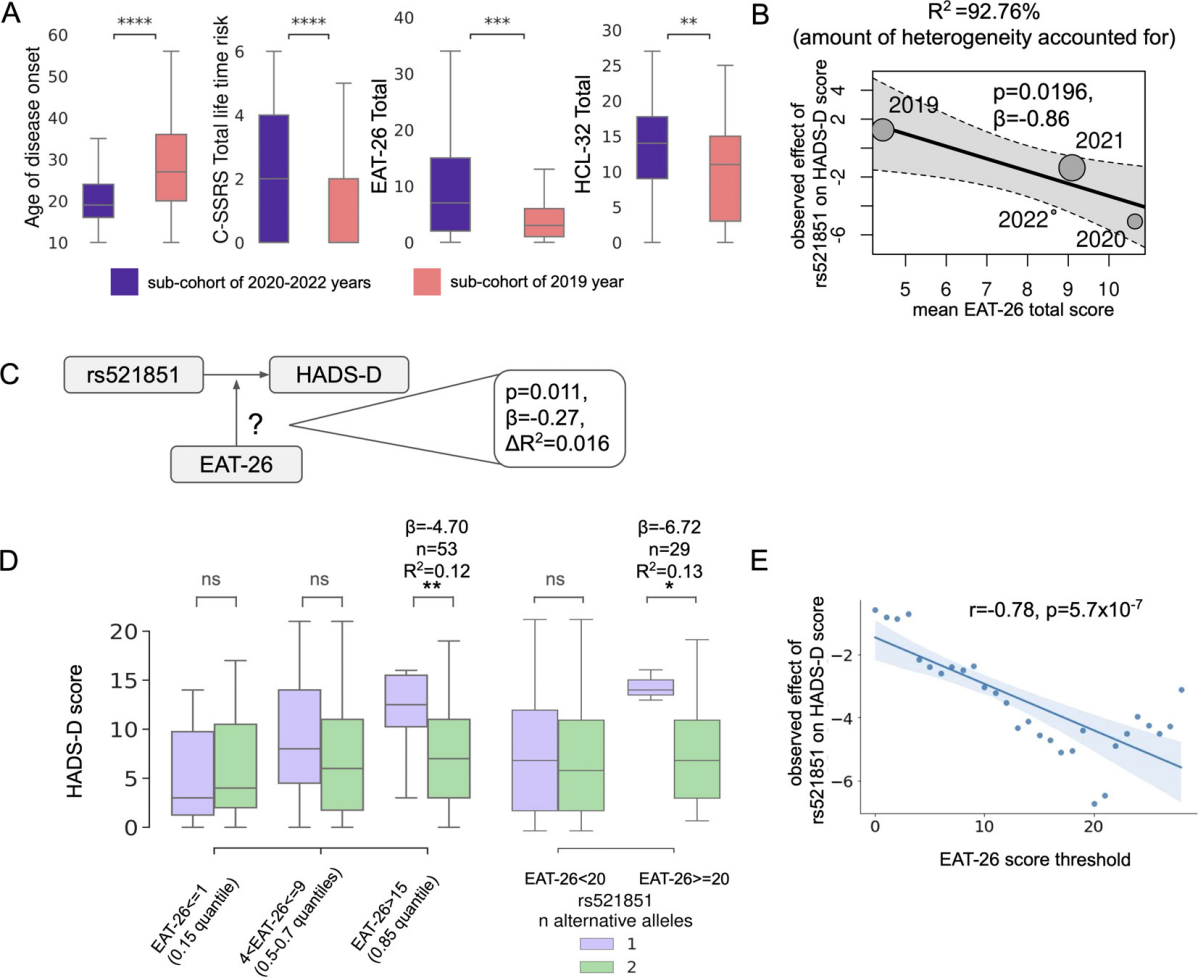
Features, which were significantly different between the 2019 and 2020–2022 sub-cohorts of patients **(A)**. Relationship between EAT-26 total scores and effect of rs521851 on HADS-D scores in patient sub-cohorts **(B)**. EAT-26 scores moderating the effect of rs521851 on HADS-D **(C)**. Changes in the effect of rs521851 genotype on HADS-D score in sub-cohorts with varying EAT-26 scores **(D)**. The absolute observed effect of rs521851 on HADS-D scores increases with the increasing EAT-26 score threshold **(E)**. *p<0.05, **p<0.01, ***p <0.001, ****p<0.0001; ns, non-significant.

In an effort to identify the sources of heterogeneity among patient sub-cohorts and explore potential moderation effects on the association of rs521851 with HADS-D score, we conducted a comprehensive comparison between the 2019 sub-cohort and the remaining patients. This comparison encompassed a variety of psychometric, anthropometric, and other features, for which responses were obtained from at least 90% of the patients (**Supplementary Table 1**). Following Bonferroni correction, seven features displayed significant differences between the 2019 and 2020–2022 patient groups. Notably, EAT dieting (p < 0.001, r = 0.94, Pearson) and EAT bulimia (p < 0.001, r = 0.72, Pearson) scores exhibited significant correlations with EAT total score, and C-SSRS lifetime suicidal ideation score demonstrated a significant (p < 0.001, r = 0.99) correlation with C-SSRS total lifetime risk score (**Supplementary Figure 1C**). Consequently, the total score features for the corresponding scales were retained for further analyses (**Figure 2A**, **Supplementary Table 1**, **Supplementary Figure 1C**).

A significant correlation was observed among all remaining features, with the highest correlation found between the EAT-26 total score and HCL-32 total score (p < 0.001, r = 0.31, Pearson). Furthermore, the EAT-26 total score exhibited a positive correlation with C-SSRS total lifetime risk score (p = 0.03, r = 0.13, Pearson) and a negative correlation with the age of disease onset (p = 0.01, r = −0.17; **Supplementary Figure 1B**). Notably, both the EAT total score and C-SSRS total lifetime risk were significantly associated with HADS-D scores (p = 0.003, beta = 0.10—t-test; and p < 0.001, beta = 0.82—t-test). Intriguingly, rs521851 did not show any significant association with these features in the studied cohort (**Supplementary Figure 1D**).

Further analysis revealed the presence of a moderation relationship between the rs521851 and EAT-26 total score in association with the HADS-D score in the entire cohort (p = 0.011—t-test, beta = −0.27, ΔR^2^ = 0.016 in the moderated regression without additional covariates). Incorporating age, sex, and research centers as covariates in the moderated regression further strengthened the evidence of interaction (p = 6 × 10^−3^—t-test, beta = −0.29, ΔR^2^ = 0.020 for the model with age and sex as additional covariates; p = 2 × 10^−3^—t-test, beta = −0.30, ΔR^2^ = 0.021 for the model with addition of research centers as covariates). EAT-26 total score explained 92.76% of the heterogeneity in the meta-analysis of patients (p = 0.0196, meta-regression; **Figures 2B, C**). The strength of the rs521851–HADS-D score association increased in subsamples of the cohort with increasing EAT scores (**Figure 2D**). The absolute effect size significantly correlated with the threshold EAT-26 score in the entire cohort (**Figure 2E**). In the sub-cohort with EAT-26 scores falling into the 0.85 quantile (EAT-26 total score > 15, n = 53), the association reached significance (p = 0.003—t-test, one sided, beta = −4.70, R^2^ = 0.12). It was also significant in the 29 participants with EAT-26 score ≥ 20 (suggestive of the presence of eating disorder risk, p = 0,0155—t-test, one sided, beta = −6.72, R^2^ = 0.13; **Figure 2D**).

In the search for the replication of the association in depression-related phenotypes in the IEU GWAS catalog, we identified the presence of the association at the p = 0.05 significance threshold for a one-sided hypothesis with probable recurrent major depression status (p = 0.048, beta = −4.37 × 10^−3^) and weight change during the worst depression episode (weight staying the same or being on a diet—p = 0.040, beta = −8.25 × 10^−3^). Additionally, the variant showed an association with a lower age at the first episode of depression (p = 0.040, beta = −0.0163). Although in the cohort considered here, the variant itself was not associated with the age of depression onset; it was significantly lower in the patient sub-cohorts of 2020–2022, in which the association was present. In the IEU GWAS, the strongest association for the variant was found with prostaglandin-H2 D-isomerase level (p = 6.92 × 10^−5^, beta = 0.16) (**Supplementary Table 3**).

## Discussion

The primary finding of the study underscores the association of the effect of rs521851 on HADS-D score with changes in eating behavior, as measured by EAT-26. This result adds substantial support to the initially reported association between the variant and HADS-D, particularly within the subgroup of patients diagnosed with ICD-10 depression.

The association between rs521851 and HADS-D scores, as discerned within the examined cohort, was predominantly influenced by patients with depression who underwent interviews during the years 2020–2022. These individuals showcased a constellation of interconnected characteristics, including an earlier age of depression onset, an elevated lifetime risk of suicide based on C-SSRS assessment, a heightened risk of eating disorders as measured by EAT-26 (particularly related to bulimia and dieting subscales), and a propensity toward hypomania, as measured by HCL-32. Subsequent analysis suggested the presence of a moderation effect of the risk of eating disorders, as measured by EAT-26, on the effect size of the rs521851–HADS-D score, indicating a particularly robust effect of the variant genotype in individuals with a high risk of eating disorders (EAT-26 score ≥ 20). Multiple lines of evidence have already linked the HADS-D depression phenotype to inflammatory bowel disease (IBD) and irritable bowel syndrome (IBS). Notably, EAT-26 scores are known to be elevated in patients with both IBS and IBD (46–48). The amplification of the association with increasing EAT-26 scores provides additional support for the involvement of the gut–brain axis mechanisms in the effect of the variant.

The most significant association of rs521851 found in IEU GWAS was that with prostaglandin-H2 D-isomerase (the product of *PTGDS*) level (p = 6.92 × 10^—5^)—a protein that catalyzes the conversion of prostaglandin H2 (PGH2) to prostaglandin D2 (PGD2) and is preferentially expressed in the brain. PGD2 is the most abundant eicosanoid in the central nervous system serving as both a neuromodulator and a trophic factor. It plays a role in the neuroinflammatory background of depression and is notably elevated under inflammatory and pathological conditions (49, 50). Decreased PGD2 levels in the brain have been observed in both MDD patients and mice exhibiting depression-like behaviors, with inhibition of its production in mice leading to increased immobility time in the forced swimming test, a behavior reversed by the antidepressant imipramine (51). Moreover, patients with MDD show higher concentrations of PGD2 in saliva compared to healthy controls (52).

In the context of gastrointestinal conditions, active Crohn’s disease patients display significantly higher levels of PGD2, and the expression of the PGD2-producing enzyme lipocalin-type prostaglandin D synthase increases in ulcerative colitis (53, 54). Exogenous prostaglandin administration can mimic many symptoms of IBS, which are alleviated by prostaglandin synthase inhibition (55). The effect of rs521851 on the interaction between the gut–brain axis and depression may involve *PTGDS*-dependent mechanisms resulting in an altered regulation of PGD2, a factor linked to both depression and gastrointestinal conditions.

We have not found direct evidence of interaction between *MAGI2* and *PTGDS*. However, as *MAGI2* is involved in regulation of the intestinal barrier permeability and functions in enteric neurons, it is plausible that rs521851, by altering *MAGI2* functionality, can be involved in gut barrier dysregulation. The resultant passage of luminal content can trigger an immunological response that promotes intestinal inflammation (56), which, in turn, is known to trigger an increase in expression of *PTGDS* and secretion of PGD2 in enteric neurons (53).

Further replication evidence from the IEU GWAS catalog indicates the association of rs521851 with an earlier age of depression onset, weight change during the worst episode of depression, and a higher likelihood of recurrent or major depression episodes. The variant is also associated with symptoms and behaviors related to irritability, appearance, and behavior, including characteristics of anxious depression, which is implicated in the emergence of suicidal tendencies (57, 58). This aligns with the observed higher co-occurrence of eating disorders and suicidal behavior, with associations found in the studied cohort between C-SSRS total lifetime risk and HADS-D score, as well as EAT total score and HADS-D score.

*MAGI2* can affect psychological symptoms of depression, measured by HADS-D scale both indirectly, through its effect on immune homeostasis associated with the gut–brain axis, as described above, and directly as a synaptic scaffolding molecule. It is localized both in excitatory and GABAergic synapses, and is important to excitation/inhibition balance. It was found to be essential for maintaining synaptic strength, enhancing AMPAR-mediated synaptic transmission, and is also involved in maintenance of GABAergic synapses (59, 60).

While replicating the original association of rs521851 with HADS-D scores among patients with clinical depression, this study underscores the variant’s role in linking the gut–brain axis with depression symptoms measured by HADS. The strength of the association is linked with alterations in eating attitudes—a factor associated with an increased risk of IBD (61) and IBS (62, 63). Lack of replication in large-scale GWAS studies of MDD may be explained by the association being linked to a specific subtype of MDD comorbid with the risk of eating disorders, characterized by a lower age of onset and higher suicidality.

Limitations of the study include a small sample size, particularly in the sub-cohort of patients with EAT scores ≥ 20, suggesting an eating disorder. Generalizability of the observed high effect of rs521851 on HADS-D scores in this patient group requires further investigation. Non-uniformity in recruitment and interview settings, except for interview year, poses another limitation. However, inclusion of research centers as an additional covariate in the moderated regression model increased the significance of the interaction term. The cross-sectional design prevents testing differences in specific trajectories of depression and/or eating disorder progression associated with rs521851.

In conclusion, the findings emphasize the importance of considering eating attitudes, suicidality, and other comorbidities when exploring the genetic basis of depression.

## Data Availability

The datasets presented in this article are not readily available because they contain personal identifiable information. Requests to access the datasets should be directed to the corresponding authors.

## References

[1] de Geus EJC. Mendelian randomization supports a causal effect of depression on cardiovascular disease as the main source of their comorbidity. J Am Heart Assoc Cardiovasc Cerebrovasc Dis. (2020) 10:e019861. doi: 10.1161/JAHA.120.019861 PMC7955496 33372533

[2] Hare DL Toukhsati SR Johansson P Jaarsma T. Depression and cardiovascular disease: a clinical review. Eur Heart J. (2014) 35:1365–72. doi: 10.1093/eurheartj/eht462 24282187

[3] Kreider KE. Diabetes distress or major depressive disorder? A practical approach to diagnosing and treating psychological comorbidities of diabetes. Diabetes Ther. (2017) 8:1–7. doi: 10.1007/s13300-017-0231-1 28160185 PMC5306125

[4] Bialek K Czarny P Strycharz J Sliwinski T. Major depressive disorders accompanying autoimmune diseases - Response to treatment. Prog Neuropsychopharmacol Biol Psychiatry. (2019) 95:109678. doi: 10.1016/j.pnpbp.2019.109678 31238086

[5] Wu Y Murray GK Byrne EM Sidorenko J Visscher PM Wray NR. GWAS of peptic ulcer disease implicates Helicobacter pylori infection, other gastrointestinal disorders and depression. Nat Commun. (2021) 12:1146. doi: 10.1038/s41467-021-21280-7 33608531 PMC7895976

[6] Pinakhina D Yermakovich D Vergasova E Kasyanov E Rukavishnikov G Rezapova V. GWAS of depression in 4,520 individuals from the Russian population highlights the role of MAGI2 (S-SCAM) in the gut-brain axis. Front Genet. (2023) 13:972196. doi: 10.3389/fgene.2022.972196 36685848 PMC9845291

[7] Hammad MM Dunn HA Ferguson SSG. MAGI proteins regulate the trafficking and signaling of corticotropin-releasing factor receptor 1 via a compensatory mechanism. J Mol Signal. (2016) 11:5. doi: 10.5334/1750-2187-11-5 31051013 PMC5345131

[8] González-Mariscal L Betanzos A Nava P Jaramillo BE. Tight junction proteins. Prog Biophys Mol Biol. (2003) 81:1–44. doi: 10.1016/S0079-6107(02)00037-8 12475568

[9] McCole DF. IBD candidate genes and intestinal barrier regulation. Inflammation Bowel Dis. (2014) 20:1829–49. doi: 10.1097/MIB.0000000000000090 PMC4357271 25215613

[10] Potkin SG Guffanti G Lakatos A Turner JA Kruggel F Fallon JH. Hippocampal atrophy as a quantitative trait in a genome-wide association study identifying novel susceptibility genes for Alzheimer’s disease. PloS One. (2009) 4:e6501. doi: 10.1371/journal.pone.0006501 19668339 PMC2719581

[11] Sapolsky RM. Depression, antidepressants, and the shrinking hippocampus. Proc Natl Acad Sci U S A. (2001) 98:12320–2. doi: 10.1073/pnas.231475998 PMC60045 11675480

[12] Opel N Redlich R Zwanzger P Grotegerd D Arolt V Heindel W. Hippocampal atrophy in major depression: a function of childhood maltreatment rather than diagnosis? Neuropsychopharmacology. (2014) 39:2723–31. doi: 10.1038/npp.2014.145 PMC4200502 24924799

[13] Santos MAO Bezerra LS Carvalho ARMR Brainer-Lima AM. Global hippocampal atrophy in major depressive disorder: a meta-analysis of magnetic resonance imaging studies. Trends Psychiatry Psychother. (2018) 40:369–78. doi: 10.1590/2237-6089-2017-0130 30234890

[14] Coleman JRI Peyrot WJ Purves KL Davis KAS Rayner C Choi SW. Genome-wide gene-environment analyses of major depressive disorder and reported lifetime traumatic experiences in UK Biobank. Mol Psychiatry. (2020) 25:1430–46. doi: 10.1038/s41380-019-0546-6 PMC7305950 31969693

[15] Brennan C Worrall-Davies A McMillan D Gilbody S House A. The Hospital Anxiety and Depression Scale: a diagnostic meta-analysis of case-finding ability. J Psychosom Res. (2010) 69:371–8. doi: 10.1016/j.jpsychores.2010.04.006 20846538

[16] McGovern DPB Taylor KD Landers C Derkowski C Dutridge D Dubinsky M. MAGI2 genetic variation and inflammatory bowel disease. Inflammation Bowel Dis. (2009) 15:75–83. doi: 10.1002/ibd.20611 PMC2614310 18720471

[17] Videlock EJ Mahurkar-Joshi S Hoffman JM Iliopoulos D Pothoulakis C Mayer EA. Sigmoid colon mucosal gene expression supports alterations of neuronal signaling in irritable bowel syndrome with constipation. Am J Physiol Gastrointest Liver Physiol. (2018) 315:G140–57. doi: 10.1152/ajpgi.00288.2017 PMC6109711 29565640

[18] Chen M Ruan G Chen L Ying S Li G Xu F. Neurotransmitter and intestinal interactions: focus on the microbiota-gut-brain axis in irritable bowel syndrome. Front Endocrinol. (2022) 13:817100/full. doi: 10.3389/fendo.2022.817100/full PMC8888441 35250873

[19] Collins SM. Interrogating the gut-brain axis in the context of inflammatory bowel disease: A translational approach. Inflammation Bowel Dis. (2020) 26:493–501. doi: 10.1093/ibd/izaa004 PMC7054772 31970390

[20] Nguyen TD Harder A Xiong Y Kowalec K Hägg S Cai N. Genetic heterogeneity and subtypes of major depression. Mol Psychiatry. (2022) 27:1667–75. doi: 10.1038/s41380-021-01413-6 PMC9106834 34997191

[21] Cai N Choi KW Fried EI. Reviewing the genetics of heterogeneity in depression: operationalizations, manifestations and etiologies. Hum Mol Genet. (2020) 29:R10–8. doi: 10.1093/hmg/ddaa115 PMC7530517 32568380

[22] Craddock N Kendler K Neale M Nurnberger J Purcell S Rietschel M. Dissecting the phenotype in genome-wide association studies of psychiatric illness. Br J Psychiatry J Ment Sci. (2009) 195:97–9. doi: 10.1192/bjp.bp.108.063156 PMC4739810 19648536

[23] Hemani G Zheng J Elsworth B Wade KH Haberland V Baird D. The MR-Base platform supports systematic causal inference across the human phenome. eLife. (2018) 7:e34408. doi: 10.7554/eLife.34408 29846171 PMC5976434

[24] Elsworth B Lyon M Alexander T Liu Y Matthews P Hallett J. MRC IEU OpenGWAS data infrastructure. bioRxiv. (2020). doi: 10.1101/2020.08.10.244293

[25] Garner DM Olmsted MP Bohr Y Garfinkel PE. The eating attitudes test: psychometric features and clinical correlates. Psychol Med. (1982) 12:871–8. doi: 10.1017/S0033291700049163 6961471

[26] Posner K Brown GK Stanley B Brent DA Yershova KV Oquendo MA. The Columbia-Suicide Severity Rating Scale: initial validity and internal consistency findings from three multisite studies with adolescents and adults. Am J Psychiatry. (2011) 168:1266–77. doi: 10.1176/appi.ajp.2011.10111704 PMC3893686 22193671

[27] Angst J Adolfsson R Benazzi F Gamma A Hantouche E Meyer TD. The HCL-32: towards a self-assessment tool for hypomanic symptoms in outpatients. J Affect Disord. (2005) 88:217–33. doi: 10.1016/j.jad.2005.05.011 16125784

[28] Cloninger CR Svrakic DM Przybeck TR. A psychobiological model of temperament and character. Arch Gen Psychiatry. (1993) 50:975–90. doi: 10.1001/archpsyc.1993.01820240059008 8250684

[29] Seabold S Perktold J. Statsmodels: econometric and statistical modeling with python. Proc 9th Python Sci Conf. (2010) 2010. doi: 10.25080/issn.2575-9752

[30] R Core Team. R: A language and environment for statistical computing. Vienna, Austria: R Foundation for Statistical Computing (2020).

[31] Viechtbauer W. Conducting meta-analyses in R with the metafor package. J Stat Software. (2010) 36:1–48. doi: 10.18637/jss.v036.i03

[32] Virtanen P Gommers R Oliphant TE Haberland M Reddy T Cournapeau D. SciPy 1.0: fundamental algorithms for scientific computing in Python. Nat Methods. (2020) 17:261–72. doi: 10.1038/s41592-019-0686-2 PMC7056644 32015543

[33] Wickham H. ggplot2: Elegant Graphics for Data Analysis. Springer-Verlag New York: Springer-Verlag (2016). Available at: https://ggplot2.tidyverse.org.

[34] Waskom ML. seaborn: statistical data visualization. J Open Source Software. (2021) 6:3021. doi: 10.21105/joss.03021

[35] Hunter JD. Matplotlib: A 2D graphics environment. Comput Sci Eng. (2007) 9:90–5. doi: 10.1109/MCSE.2007.55

[36] Charlier F Weber M Izak D Harkin E Magnus M Lalli J. Statannotations (v0.6) (2023). Available online at: https://zenodo.org/records/8396665.

[37] Fedor. gecko984/supervenn (2024). Available online at: https://github.com/gecko984/supervenn.

[38] Reback J McKinney W jbrockmendel den Bossche JV Augspurger T Cloud P. pandas-dev/pandas: Pandas 1.0.3. Zenodo (2020). Available at: https://zenodo.org/record/3715232.

[39] Harris CR Millman KJ van der Walt SJ Gommers R Virtanen P Cournapeau D. Array programming with numPy. Nature. (2020) 585:357–62. doi: 10.1038/s41586-020-2649-2 PMC7759461 32939066

[40] Wickham H Averick M Bryan J Chang W McGowan LD François R. Welcome to the tidyverse. J Open Source Software. (2019) 4:1686. doi: 10.21105/joss.01686

[41] Wickham H François R Henry L Müller K. dplyr: A Grammar of Data Manipulation. R package version 1.0.2 (2020). Available online at: https://CRAN.R-project.org/package=dplyr.

[42] Hail Team. Hail o.2 . Available online at: https://github.com/hail-is/hail.

[43] Genovese G. gtc2vcf (2023). Available online at: https://github.com/freeseek/gtc2vcf.

[44] Browning BL Zhou Y Browning SR. A one-penny imputed genome from next-generation reference panels. Am J Hum Genet. (2018) 103:338–48. doi: 10.1016/j.ajhg.2018.07.015 PMC6128308 30100085

[45] Zigmond AS Snaith RP. The hospital anxiety and depression scale. Acta Psychiatr Scand. (1983) 67:361–70. doi: 10.1111/j.1600-0447.1983.tb09716.x 6880820

[46] Wabich J Bellaguarda E Joyce C Keefer L Kinsinger S. Disordered eating, body dissatisfaction, and psychological distress in patients with inflammatory bowel disease (IBD). J Clin Psychol Med Settings. (2020) 27:310–7. doi: 10.1007/s10880-020-09710-y 32172438

[47] Kayar Y Agin M Dertli R Kurtulmus A Boyraz RK Onur NS. Eating disorders in patients with irritable bowel syndrome. Gastroenterol Hepatol. (2020) 43:607–13. doi: 10.1016/j.gastrohep.2020.03.001 32718838

[48] Evans KM Averill MM Harris CL. Disordered eating and eating competence in members of online irritable bowel syndrome support groups. Neurogastroenterol Motil. (2023) 35:e14584. doi: 10.1111/nmo.14584 36989182 PMC10524246

[49] Regulska M Szuster-Głuszczak M Trojan E Leśkiewicz M Basta-Kaim A. The emerging role of the double-edged impact of arachidonic acid-derived eicosanoids in the neuroinflammatory background of depression. Curr Neuropharmacol. (2021) 19:278–93. doi: 10.2174/18756190MTA4dOTMh0 PMC8033972 32851950

[50] Zhang S Grabauskas G Wu X Joo MK Heldsinger A Song I. Role of prostaglandin D2 in mast cell activation-induced sensitization of esophageal vagal afferents. Am J Physiol Gastrointest Liver Physiol. (2013) 304:G908–916. doi: 10.1152/ajpgi.00448.2012 PMC3652067 23471341

[51] Chu C Wei H Zhu W Shen Y Xu Q. Decreased prostaglandin D2 levels in major depressive disorder are associated with depression-like behaviors. Int J Neuropsychopharmacol. (2017) 20:731–9. doi: 10.1093/ijnp/pyx044 PMC5581486 28582515

[52] Ohishi K Ueno R Nishino S Sakai T Hayaishi O. Increased level of salivary prostaglandins in patients with major depression. Biol Psychiatry. (1988) 23:326–34. doi: 10.1016/0006-3223(88)90283-1 3422573

[53] Le Loupp AG Bach-Ngohou K Bourreille A Boudin H Rolli-Derkinderen M Denis MG. Activation of the prostaglandin D2 metabolic pathway in Crohn’s disease: involvement of the enteric nervous system. BMC Gastroenterol. (2015) 15:112. doi: 10.1186/s12876-015-0338-7 26338799 PMC4558965

[54] Sturm EM Radnai B Jandl K Stančić A Parzmair GP Högenauer C. Opposing roles of Prostaglandin D2 receptors in ulcerative colitis. J Immunol Baltim Md 1950. (2014) 193:827–39. doi: 10.4049/jimmunol.1303484 PMC4121674 24929001

[55] Hasler WL Grabauskas G Singh P Owyang C. Mast cell mediation of visceral sensation and permeability in irritable bowel syndrome. Neurogastroenterol Motil. (2022) 34:e14339. doi: 10.1111/nmo.14339 35315179 PMC9286860

[56] Michielan A D’Incà R. Intestinal permeability in inflammatory bowel disease: pathogenesis, clinical evaluation, and therapy of leaky gut. Mediators Inflamm. (2015) 2015:628157. doi: 10.1155/2015/628157 26582965 PMC4637104

[57] Fava M Alpert JE Carmin CN Wisniewski SR Trivedi MH Biggs MM. Clinical correlates and symptom patterns of anxious depression among patients with major depressive disorder in STAR*D. Psychol Med. (2004) 34:1299–308. doi: 10.1017/S0033291704002612 15697056

[58] Coren S Hewitt PL. Is anorexia nervosa associated with elevated rates of suicide? Am J Public Health. (1998) 88:1206–7. doi: 10.2105/ajph.88.8.1206 PMC1508312 9702149

[59] Danielson E Zhang N Metallo J Kaleka K Shin SM Gerges N. S-SCAM/MAGI-2 is an essential synaptic scaffolding molecule for the gluA2-containing maintenance pool of AMPA receptors. J Neurosci. (2012) 32:6967–80. doi: 10.1523/JNEUROSCI.0025-12.2012 PMC3365591 22593065

[60] Shin SM Skaar S Danielson E Lee SH. Aberrant expression of S-SCAM causes the loss of GABAergic synapses in hippocampal neurons. Sci Rep. (2020) 10:83. doi: 10.1038/s41598-019-57053-y 31919468 PMC6952429

[61] Day AS Yao CK Costello SP Andrews JM Bryant RV. Food avoidance, restrictive eating behaviour and association with quality of life in adults with inflammatory bowel disease: A systematic scoping review. Appetite. (2021) 167:105650. doi: 10.1016/j.appet.2021.105650 34391842

[62] Jia W Liang H Wang L Sun M Xie X Gao J. Associations between abnormal eating styles and irritable bowel syndrome: A cross-sectional study among medical school students. Nutrients. (2022) 14:2828. doi: 10.3390/nu14142828 35889787 PMC9319336

[63] Guo YB Zhuang KM Kuang L Zhan Q Wang XF Liu SD. Association between diet and lifestyle habits and irritable bowel syndrome: A case-control study. Gut Liver. (2015) 9:649–56. doi: 10.5009/gnl13437 PMC4562783 25266811

