# Supplementary material for: The effect size of rs521851 in the intron of MAGI2/S-SCAM on HADS-D scores correlates with EAT-26 scores for eating disorders risk
Source: Front Psychiatry. 2024 Dec 5;15:1416009. doi: 10.3389/fpsyt.2024.1416009 (PMC11656592; doi:10.3389/fpsyt.2024.1416009)
Supplement: Supplementary file 1 [file DataSheet1.pdf]

Effect size of rs521851 in the intron of MAGI2/S-SCAM on HADS-D scores correlates with eating behaviours changes

Supplementary Data

Research Centers

1. V.M. Bekhterev National Medical Research Center for Psychiatry and Neurology (Saint Petersburg), Mental Health Research Center (Moscow),
2. Peoples' Friendship University of Russia, Rostov State Medical University (Rostov-on-Don),
3. Institute of Biochemistry and Genetics of Ufa Federal Research Center of the Russian Academy of Sciences (Ufa),
4. Serbsky National Medical Research Centre on Psychiatry and Addictions (Moscow),
5. Lipetsk Regional Addiction Hospital, (Lipetsk),
6. Moscow Scientific and Practical Center for Narcology,
7. Psychiatric clinical hospital #4 named after P.B. Gannushkin,
8. Ryazan State Medical University named after academician I.P. Pavlov,
9. Psychiatric clinical hospital No. 13 (Moscow)

Psychometric Research Tools

To be completed by a physician:

1) Columbia Suicide Severity Rating Scale (C-SSRS) - to assess the risk of suicide (Oquendo M.A., Halberstam B., 2003).

For self-report by participants:

2) Cloninger scale for assessing personality traits, character and temperament (Temperament Character Inventory, TCI) (Cloninger C.R., 1994). The version of the questionnaire adapted in the Russian Federation will be used (Enikolopov S.N.,Efremov A.G., 2001, 2002);

3) Hospital Anxiety and Depression Scale (HADS) to assess the severity of affective disorders (Zigmond A.S, Snaith R.P., 1983);

4) Hypomania questionnaire (HCL-32) to exclude patients with latent (hypo-) manic (Angst J., Adolfsson R., Benazzi F. et al.,2005);

5) Eating Attitude Test (EAT-26) to assess the risk of eating disorders (Garner D.M. et al., 1982);

6) Snaith-Hamilton Anhedonia Scale (SHAPS) to assess the severity of anhedonia (Snaith R.P., Hamilton M., Morley S.,Humayan A., 1995).

Exclusion Criteria

All participants signed an informed consent before recruitment in the study and were required to be aged 18 years or older.

Excluded from the study were patients with bipolar disorder (BD), schizophrenic spectrum disorders, organic mental disorders, a history of seizure syndrome, or severe decompensated diseases, including cardiovascular, neurological, endocrine, hematological diseases, liver, kidney, gastrointestinal tract, respiratory system, and thyroid diseases. Exclusion criteria also encompassed patient refusal to participate in the study and the care provider's decision to exclude patients openly exhibiting aggressive behavior or posing a threat to themselves or others.

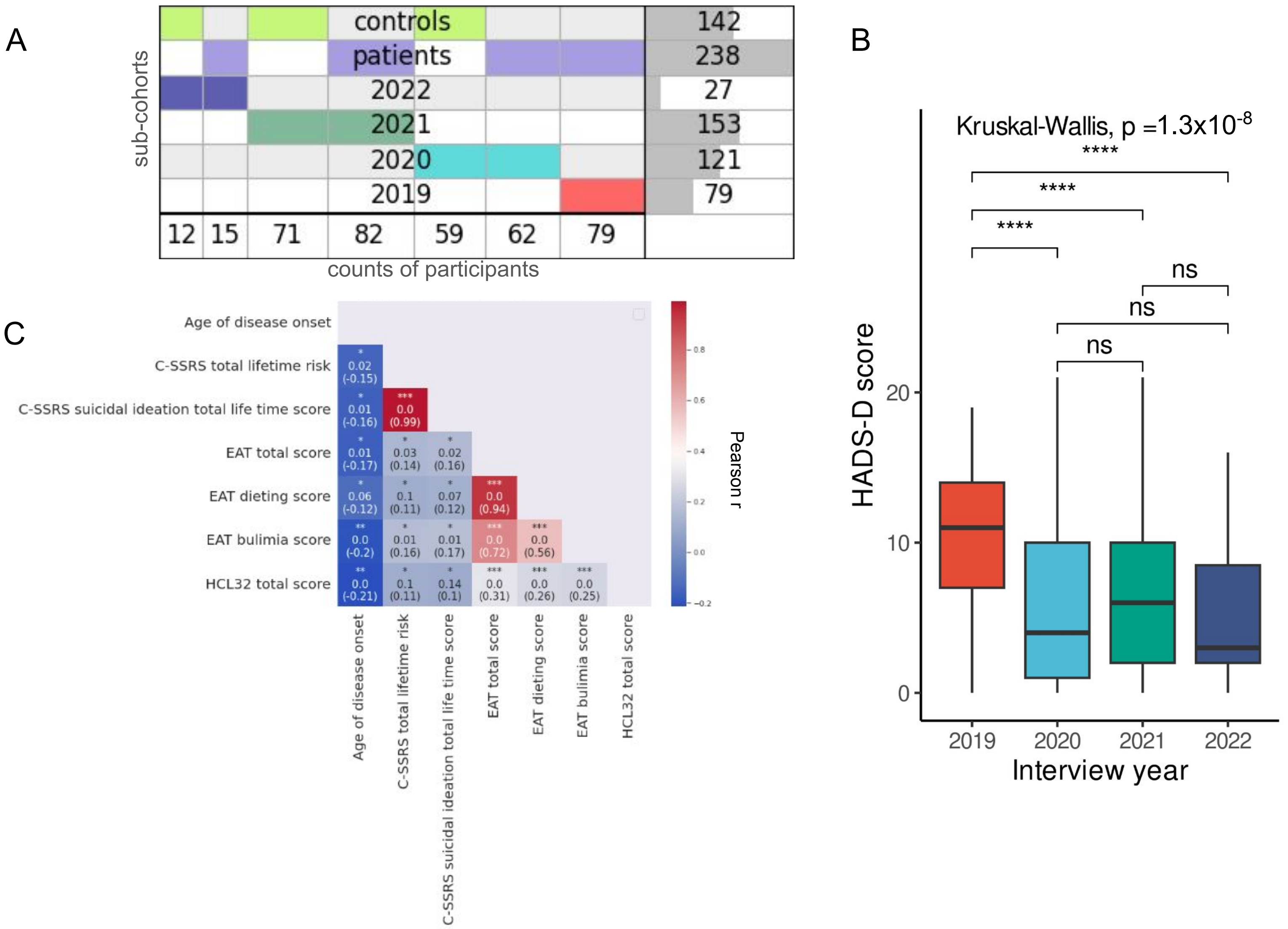

D

Feature (n total participants)	Mean, SE: Patients 2019	Mean, SE: Patients 2020-2022	MW p-value (adj.)	HADS-D score association (OLS regression)	rs521851 association (OLS regression)	Significance of interaction with rs52185 in HADS-D score prediction (moderated regression)
Age of disease onset (n=238)	**28.84, SE=1.33**	**20.97, SE=0.60**	8.45×10^{-7}	p=0.611	p=0.464	p=0.270
C-SSRS total lifetime risk (n=366)	**1.08, SE=0.21**	**2.32, SE=0.17**	8.42×10^{-6}	p=0.000, beta=0.82	p=0.723	p=0.104
EAT total score (n=379)	**4.86, SE=0.61**	**9.60, SE=0.73**	2.88×10^{-3}	p=0.003, beta=0.10	p=0.749	p=0.011, beta=-0.27, ΔR^2=0.016
HCL32 total score (n=230)	**10.10, SE=0.87**	**13.34, SE=0.53**	3.36×10^{-2}	p=0.545	p=0.503	p=0.680

Fig. S1. Sub-cohort composition (A). Differences in HADS-D scores between sub-cohorts of 2019-2022 years (B). Relationship between HADS-D phenotypes and interview year in the whole cohort (2019-2022) and in the sub-cohort of 2020-2022 (C). Correlation (Pearson) between features which are significantly different between 2019 and 2020-2022 sub-cohorts of patients, corresponding p-values and coefficients (in parentheses) (D). Main features, significantly different between the 2019 and 2020-2022 sub-cohorts of patients, their values in the sub-cohorts, results of Mann-Whitney test, association with HADS-D scores and rs521851, significance of interaction with rs52185 in prediction of HADS-D scores in the entire cohort.

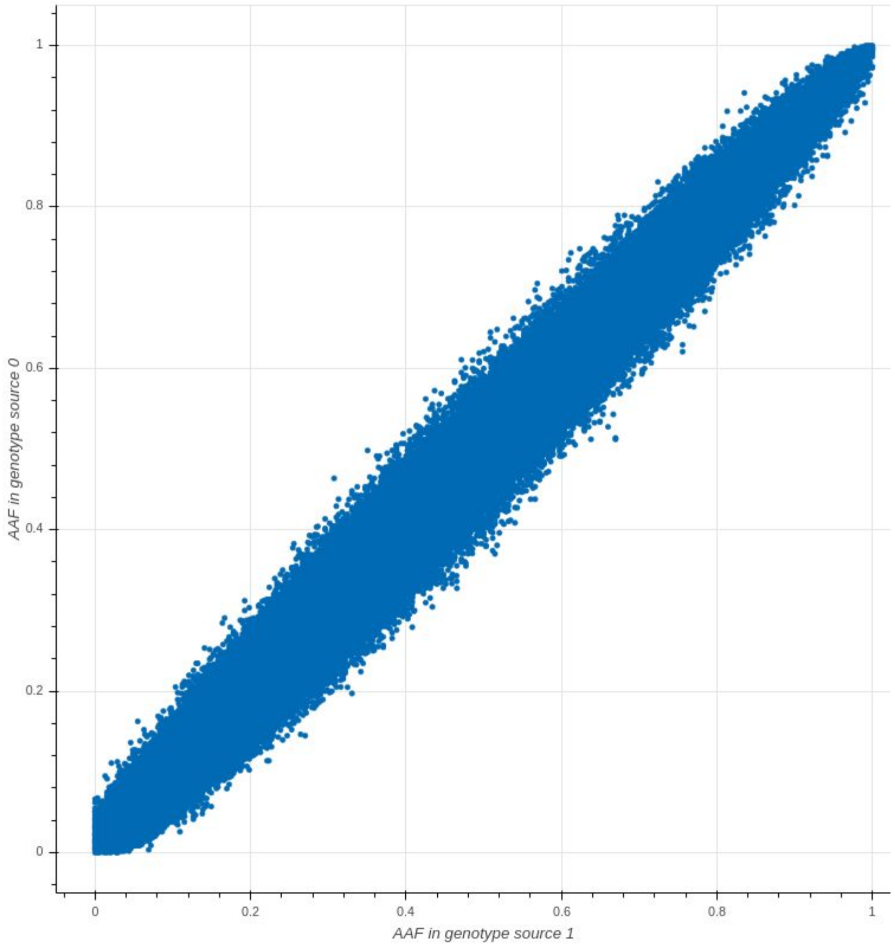

Fig. S2. Comparison between alternative allele frequencies in the samples from the two genotyping runs.

Table S1. Comparison between 2019 and 2022-2022 sub-cohorts of patients across the studied features. pvalue_MW - p-value for a Mann-Whitney test, n - total count of patients, for which the feature was evaluated.

features	pvalue_MW	n	mean_2019	mean_2020_2022	se_mean_2019	se_mean_other	pvalue_MW_Bonferroni_corrected
Age_of_disease_onset	2.224485e-08	238	28.8354430	20.9685535	1.32871201	0.59550243	8.675490e-07
C-SSRS_RISK_TOTAL_LIFE_TIME	2.216788e-07	236	1.0779221	2.3207547	0.21140276	0.16960332	8.645472e-06
C-SSRS_SUICIDAL_IDEATION_TOTAL_LIFE_TIME	2.878796e-07	236	1.0000000	2.1320755	0.19389168	0.14799383	1.122730e-05
EAT_total	7.569973e-05	237	4.8589744	9.6037736	0.61035693	0.72618734	2.952290e-03
EAT_dieting	3.864912e-04	237	3.0769231	6.4591195	0.46192680	0.53607553	1.507316e-02
EAT_Bulimia_Food_Preoccupation	6.206454e-04	238	0.2784810	1.2075472	0.07839826	0.19117449	2.420517e-02
HCL32_total	8.846748e-04	228	10.1000000	13.3417722	0.87456585	0.53414963	3.450232e-02
C-SSRS_RISK_CATEGORY_LIFETIME	1.341755e-03	236	1.3636364	1.7044025	0.08453380	0.07056893	5.232843e-02
Waist_circumpherence	1.430536e-03	231	82.4430380	76.3815789	1.77524421	1.09188897	5.579091e-02
TCI_novelty	2.224048e-03	233	7.3333333	8.6329114	0.36234140	0.25733682	8.673787e-02
Education_level_full	5.239325e-03	238	3.4050633	3.0943396	0.10887133	0.08360196	2.043337e-01
Education_length	1.007018e-02	238	12.2405063	14.5597484	0.57965484	0.21443624	3.927372e-01
TCI_transcend	1.183497e-02	232	4.0266667	5.1592357	0.32658392	0.27845341	4.615637e-01
Weight_kg	1.875743e-02	238	72.4822785	67.1163522	1.98870697	1.12413396	7.315399e-01
C-SSRS_SUICIDAL_BEHAVIOR_TOTAL_LIFE_TIME	1.960229e-02	236	0.4545455	0.8553459	0.13272662	0.11998609	7.644892e-01
HADS_anxiety	2.019206e-02	237	11.3797468	9.2341772	0.69991046	0.32575587	7.874904e-01
BWI	2.141687e-02	238	24.9388608	23.3488050	0.65709176	0.34599076	8.352578e-01
CTQ_Emotional_Abuse	2.569866e-02	231	8.8533333	10.5256410	0.46137020	0.42419083	1.002248e+00
TCI_self	2.844499e-02	233	13.6266667	12.3670886	0.55337566	0.39399614	1.109355e+00
TCI_persistence	2.935866e-02	233	1.9866667	2.3227848	0.12943861	0.10710056	1.144988e+00
CTQ_Sexual_Abuse	4.356224e-02	234	5.1600000	5.6415094	0.07361943	0.16555629	1.698928e+00
Count_of_serious_suicidal_attempts	4.829141e-02	238	0.2151899	0.4213836	0.05602570	0.06579942	1.883365e+00
CTQ_Minimization_Denial	5.794863e-02	233	9.2533333	8.5253165	0.35468211	0.28701077	2.259997e+00
HADS_anxiety_category	7.345117e-02	237	2.2278481	2.0632911	0.09859343	0.06760287	2.864596e+00
Typical_count_of_cigarettes_per_day_when_smoking_regularly	7.915922e-02	236	4.5714286	4.4716981	0.93453315	0.52193440	3.087210e+00
CTQ_Physical_Abuse	9.931479e-02	234	6.0933333	6.8679245	0.23262957	0.28047312	3.873277e+00
SHAPS_total	1.232797e-01	233	5.2894737	4.7452229	0.41371903	0.31027171	4.807907e+00
EAT_Oral_Control	1.292911e-01	238	1.4810127	1.9371069	0.21306008	0.19130147	5.042353e+00
CTQ_Total_score	1.395558e-01	231	40.4933333	43.3910256	1.32873622	1.16378258	5.442678e+00
Height	1.541528e-01	238	170.5063291	169.3333333	1.12304841	0.64431934	6.011958e+00
Suicidal_attempt_severity	1.655204e-01	238	0.7215190	0.7610063	0.17919789	0.11886036	6.455297e+00
CTQ_Minimization_Denial_Rate	1.922453e-01	233	0.4933333	0.4430380	0.09931900	0.07041284	7.497567e+00
CTQ_Physical_Neglect	2.345285e-01	234	8.1866667	7.9937107	0.32917770	0.23121174	9.146610e+00
CTQ_Emotional_Neglect	2.532869e-01	233	12.2000000	12.6708861	0.61921194	0.43188292	9.878191e+00
TCI_coop	2.789299e-01	230	17.5466667	17.0000000	0.52066947	0.38921359	1.087826e+01
TCI_reward	4.172197e-01	233	8.2133333	8.2911392	0.34342910	0.25098432	1.627157e+01
Length_of_the_longest_period_of_depression	4.229871e-01	237	18.5897436	13.8930818	5.83336243	3.18378724	1.649650e+01
Education_level_short	4.601811e-01	238	1.7341772	1.6855346	0.05613559	0.04898327	1.794706e+01
TCI_harm	4.660063e-01	233	14.7631579	14.4713376	0.41458854	0.34416825	1.817425e+01

Table S2. Association between rs521851 and HADS-D phenotypes across the studied sub-cohorts.

Clinical depression status	HADS-D phenotype definition	Test (one-sided)	**Sub-cohorts** (n of patient (p) and healthy (h) participants)			
			**2019** n(p)=79 n(h)=0	**2020** n(p)=62 n(h)=59	**2021** n(p)=82 n(h)=71	**2022** n(p)=15 n(h)=12
**Mixed** (n=380)	HADS status (score>=11)	Fisher exact	OR=1.49, p=0.838	OR=0.55, p=0.206	OR=0.57 p=0.172	OR=0.08, p=0.079
		Mantel-Haenszel		OR=0.49, p=0.026, I^2=4.64%, Q test p=0.350 (n=301)		
			OR=0.66, p=0.103, I^2=37.29%, Q test p=0.189			
	HADS score	t-test	beta=1.26, p=0.194	beta=-0.64, p=0.323	beta=-0.59, p=0.276	beta=-5.87, p=0.020
		t-test, FE		beta=-1.03, p=0.090, I^2=41.81%, Q test p=0.179		
			beta=-0.53, p=0.221, I^2=44.25%, Q test p=0.146			
**Patients** (n=238)	HADS status (score>=11)	Fisher exact	OR=1.49, p=0.838 (n=79)	OR=0.11, p=0.022 (n=62)	OR=0.50, p=0.164 (n=82)	OR=0.12, p=0.154 (n=15)
		Mantel-Haenszel		OR=0.30, p=$5x10^{-3}$, I^2=14.87%, Q test p=0.310		
			OR=0.54, p=0.048, I^2=54.30%, Q test p=0.087			
	HADS score	t-test	beta=1.26, p=0.194	beta=-5.08, p=$5x10^{-3}$	beta=-1.36, p=0.143	beta=-4.42, p=0.098
		t-test, FE		beta=-2.68, p=$3.8x10^{-3}$, I^2=31.95%, Q test p=0.230		
			beta=-1.40, p=0.045, I^2=62.14%, Q test p=0.048			
**Healthy participants** (n=142)	HADS status (score>=11)	Fisher exact		OR=1.03, p=0.683*		
	HADS score	t-test		beta=1.02, p=0.926		

significant results, the true outcomes do not appear significantly heterogeneous according to the Q test

significant results, the true outcomes appear heterogeneous according to the Q test

a effect direction replicates the original study

a effect direction does not replicate the original study

* 1 is added to all elements in the contingency table due to absence of cases with a single alternative allele.

Table S3. rs521851 association with a range of other features based on a phewas with IEU OpenGWAS data.

**Trait (IEU GWAS ID)**	**p**	**beta**	**se**
Prostaglandin-H2 D-isomerase (prot-a-2426)	6.92×10^{-5}	3	0.040
Bipolar and major depression status: Probable Recurrent major depression (moderate) (ukb-d-20126_4)	0.048 (one-sided)	-4.37×10^{-3}	2.63×10^{-3}
Weight change during worst episode of depression: Stayed about the same or was on a diet (ukb-d-20536_0)	0.040 (one-sided)	-8.25×10^{-3}	4.74×10^{-3}
Age at first episode of depression (ukb-d-20433_irnt)	0.040 (one-sided)	-0.0163	9.32×10^{-3}
Longest period of depression (ukb-e-4609_AFR)	7.98×10^{-3}	0.12	0.05
Feelings of tiredness during worst episode of depression (ukb-d-20449)	9.86×10^{-3}	9.50×10^{-3}	3.68×10^{-3}
Depression possibly related to stressful or traumatic event (ukb-d-20447) (was asked to participants with signs of depression)	0.034	8.54×10^{-3}	4.03×10^{-3}
Difficulty concentrating during worst depression (ukb-d-20435)	0.039	8.19×10^{-3}	3.96×10^{-3}
Weight change during worst episode of depression: Both gained and lost some weight during the episode (ukb-d-20536_3)	0.046	4.84×10^{-3}	2.43×10^{-3}
Symptoms and signs involving appearance and behaviour (finn-b-R18_SYMPTOMS_SIGNS_INVOLVI_APPEA_BEHVI)	0.013 (one-sided)	-0.64	0.287
Irritability (ukb-e-1940_MID)	0.010 (one-sided)	-0.29	0.124

the most associated phenotype in the Phewas

phenotypes related to depression with the same effect direction as the analysed association

phenotypes related to depression

other phenotypes
